# Evaluation of a hospital-based opioid stewardship program on high-risk opioid prescribing in a Canadian setting: an interrupted time series analysis

**DOI:** 10.1186/s13722-025-00574-x

**Published:** 2025-06-06

**Authors:** Lianping Ti, Tamara Mihic, Arielle Beauchesne, Cameron Grant, Ingrid Frank, Nooreen Haji, Michael Legal, Stephen Shalansky, Seonaid Nolan

**Affiliations:** 1 British Columbia Centre on Substance Use, 400-1045 Howe Street, Vancouver, BC V6Z 2A9 Canada; 2 Department of Medicine, University of British Columbia, 400-1045 Howe Street, Vancouver, BC V6H 0A5 Canada; 3 Faculty of Pharmaceutical Sciences, University of British Columbia, 2405 Wesbrook Mall, Vancouver, BC V6T 2A1 Canada; 4 Pharmacy Department, Providence Health Care, 1081 Burrard Street, Vancouver, BC V6Z 1Y6 Canada; 5 Pharmacy Department, Victoria General Hospital, 1 Hospital Way, Victoria, BC V8Z 6R5 Canada; 6 Pharmacy Department, Surrey Memorial Hospital Pharmacy, 13750 96th Avenue, Surrey, BC V3V 1Z2 Canada

**Keywords:** Opioid stewardship program, Evaluation, Hospital, High-risk prescribing

## Abstract

**Background:**

High-risk opioid prescribing (e.g., high daily dose opioids, concurrent opioid-sedatives) is prevalent in hospitals and linked to adverse outcomes. Opioid stewardship programs (OSP) have the potential to reduce high-risk opioid prescribing through audit-and-feedback recommendations.

**Methods:**

We evaluated an audit-and-feedback based OSP implemented in January 2020 at a Vancouver, Canada tertiary care hospital using interrupted time series analysis. An electronic health record (EHR) system with computerized provider order entry (CPOE) was simultaneously operationalized. The main outcome was: any high-risk opioid prescribing (based on 10 evidence-based indicators), including high daily dose of morphine milligram equivalent (MME) prescribing (> 90MME), long opioid prescription duration (> 5 days post-admission), and concurrent opioid-sedative prescribing.

**Results:**

Between January 2018 and March 2022, 5,477 active opioid patient encounters were included. While no significant change occurred in overall high-risk opioid prescribing post-OSP (*p* > 0.05), a significant reduction was seen in the level of high daily dose of MME prescriptions (estimate: -0.044; 95% confidence interval [CI]: -0.082, -0.006). Conversely, the trend in long opioid duration increased (estimate: 0.006; 95%CI: 0.000, 0.011), likely due to the removal of automatic stop dates with the implementation of the EHR with CPOE. Post-OSP intervention, we initially saw an acute increase in concurrent opioid-sedative prescriptions (estimate: 0.013; 95%CI: 0.005, 0.020). A benzodiazepine ordering intervention implemented in May 2021 reversed this trend, reducing both the level (estimate: 0.874; 95%CI: 0.374, 1.375) and slope (estimate: -0.022, 95%CI: -0.034, -0.011) of concurrent prescriptions.

**Conclusion:**

The implementation of a new EHR concordant with that of the OSP may have impacted our study’s results. While our research suggests the OSP reduced high-dose opioid prescribing, other indicators impacted by the EHR system did not benefit as highly from the OSP. Nevertheless, the OSP proved able to rapidly respond to unintended consequences by introducing interventions to reduce concurrent opioid and sedative prescribing.

**Supplementary Information:**

The online version contains supplementary material available at 10.1186/s13722-025-00574-x.

**What is already known on the topic**:

- Audit-and-feedback interventions involving pharmacists are effective as a quality and safety control strategy for changing prescribing patterns among prescribers.
- Opioid stewardship programs have been shown to reduce the amount of opioid prescribing in postsurgical and emergency department settings.

**What this study adds**:

- A hospital-based opioid stewardship program immediately reduced the proportion of high daily dose opioid prescribing but negatively impacted some high-risk opioid prescribing outcomes that were susceptible to changes to computerized order systems (i.e., long duration of prescribing and concurrent opioid-sedative prescribing).
- Monitoring through the opioid stewardship program allowed the clinical team to respond and intervene on high-risk prescribing patterns introduced by the electronic system.

**How this study might affect research**,** practice or policy**:

- Opioid stewardship programs that incorporate audit-and-feedback mechanisms alongside consultations have the potential to be used as a quality and safety monitoring tool to minimize exposure to high-risk opioids in a hospital setting.

## Introduction

North America has been disproportionately affected by an ongoing public health crisis originating in part from the overprescribing of opioid medications [1, 2]. The United States and Canada are among the highest consumers of opioids globally, and in 2018, approximately 1 in 7 Canadians were being prescribed opioids [3–5]. Of particular concern is the variability in opioid prescribing practices in acute care settings that can increase the risk of adverse events, including high daily dose of opioids at levels above clinical recommendations, prescription of multiple opioids, long duration of opioid prescribing, and concurrent opioid-sedative prescribing [6–8]. Data from community dispensations in Ontario, Canada, indicate that in 2019, 1 in 7 patients newly treated with opioids for pain were receiving the medication for more than 7 days, and 1 in 5 patients were receiving an initial daily dose above 50 morphine milligram equivalents (MME), which has shown to increase risk of negative health impacts [9]. While cutoffs to define high dose MME vary across settings, clinical guidelines have suggested that amounts beyond 50MME are more likely to yield poorer benefits for pain relative to the risks [10]. Corresponding with high rates of opioid prescribing has been the development of opioid misuse, addiction and prescription opioid-related overdose deaths, as well as other related morbidities, including opioid-induced hyperalgesia and sleep-disordered breathing [8–11]. 

Initial exposure to prescription opioids and continued use, sometimes at high doses, is common in hospital settings, particularly for acute pain management following surgical interventions [11, 12]. While hospitals provide essential care for patients, studies have shown that it can also act as a risk environment that contributes to the opioid epidemic and related harms [13]. A study of 286 US hospitals found that the majority of hospitalized patients were exposed to high doses of opioid analgesics (i.e., daily doses of > 100MME) and that hospitals that use opioids most frequently had increased rates of severe opioid-related adverse drug events [14]. Other studies indicate that prescribing-related factors such as concurrent use of opioids and sedatives and multiple opioid prescriptions on the same day were associated with opioid-related adverse drug events among hospitalized patients [15–17]. Moreover, past research has shown that short-term opioid use among post-surgical and opioid naïve patients during hospitalization can lead to prolonged use and subsequent opioid use disorder [11, 12, 18, 19]. While opioids play a critical role in managing acute pain in hospitals, careful consideration of their risks and benefits is essential to prevent unintended consequences associated with prescribing opioids for pain.

Systems-level interventions such as opioid stewardship programs have the potential to optimize prescribing practices while maintaining or improving pain management for patients receiving opioids during their acute admission [20]. While current definitions and program models vary, the concept of opioid stewardship has been largely based on antimicrobial stewardship [21–23]. These programs are typically multidisciplinary-led by pharmacists and physicians and involve a prospective screening process, formulary restrictions, and/or educational activities [20, 24]. Previous studies have shown that incorporation of audit-and-feedback strategies within opioid stewardship reduced the amount of opioid prescribing among clinicians in postsurgical and emergency departments [25, 26]. A smaller number of preliminary studies in hospital settings have shown cost-savings, a reduction in opioid-associated rapid response calls and cardiorespiratory arrests, and successful interventions related to pain medication reconciliation [27, 28]. While there is growing research in this area, an online survey of hospitals globally conducted in 2018 indicated only 23% reported a stewardship program, and even less (14%) reported the use of a prospective screening process [20]. 

As described, most studies have previously focused on the impact of opioid stewardship on the amount of opioid prescribing, despite knowing that a reduction in opioid use is not always the goal for optimizing pain management and improving patient safety. Little is known about the impact of hospital-based opioid stewardship programs on other modifiable prescribing indicators associated with increased risk of adverse events, or long-term use and dependence. The objective of this study was to evaluate the impact of an audit-and-feedback based opioid stewardship program on various high-risk opioid prescribing practices (e.g., long duration of opioid prescriptions, concurrent opioid-sedating prescriptions) in an acute care setting in Vancouver, Canada.

## Methods

### Study setting

The present study took place at St. Paul’s Hospital, which is located in downtown Vancouver. St. Paul’s Hospital is a 400-bed acute care teaching hospital and is the provincial referral centre for a number of specialty surgical services, including cardiac surgery and gastrointestinal surgery. The hospital also offers various consulting services that are dedicated to managing pain and addiction. These include an interdisciplinary addiction medicine consult service, acute and complex pain services, and palliative care service.

In November 2019, Cerner, an electronic health record system (EHR) with computerized provider order entry (CPOE) system, was gradually rolled-out at St. Paul’s Hospital as well as other sites across the Lower Mainland region of British Columbia in an effort to provide more real-time data and consistent care to patients [29]. As part of the Cerner implementation, and relevant to this study, automatic stop dates were removed for opioid and benzodiazepine orders. Prior to Cerner implementation, orders would automatically be discontinued after 7 days for opioids and 30 days for benzodiazepines if a continuation order was not written by the prescriber. The Cerner system also allowed for the ordering of benzodiazepines days before short procedures (e.g., insertion of a peripherally inserted central catheter line, endoscopy, and bronchoscopy) with unclear discontinuation plans, a capability that was previously not possible.

Shortly after, in January 2020, Vancouver’s first opioid stewardship program was implemented at St. Paul’s Hospital. The opioid stewardship program is operated by a full-time clinical pharmacy specialist and a part-time addiction medicine physician, who were hired specifically with protected time to monitor active opioid prescriptions and provide audit-and-feedback recommendations, consultations, and educational activities related to the program and in-hospital opioid prescribing [31]. The audit-and-feedback component utilizes an automated screening algorithm as an initial screening approach to assist the clinical team in identifying potential high-risk opioid prescribing indicators. Shown in Table 1, thirteen patient- and prescribing-related indicators were adapted based on national and international clinical guidelines, research articles, and those developed by healthcare providers with pain-related expertise [6, 20, 30, 31], as well as availability of data elements in the pharmacy system used to generate the screening report. A shortlist of patients admitted to inpatient units, excluding the hospital’s critical care units or emergency department, and prescribed at least one opioid is generated. Following initial screening, those seen by the addiction medicine, palliative care, or the acute and complex pain services are excluded due to their distinct opioid needs and higher risks compared to the general population. Subsequently, remaining patients with the highest number of outcome indicators are selected to receive a full assessment by the clinical team. This approach allowed the opioid stewardship team to prioritize those in most need given capacity and time constraints with not being able to intervene on all patients who are at risk, and aims to optimize analgesic therapy and enhance pain management while prioritizing patient safety. The opioid stewardship team followed standard clinical pharmacy assessment by reviewing the patient’s chart and engaging in discussions with both the patient and primary care team. Any recommendations are communicated to the patient’s most responsible care team (through Cerner or verbally), documented in a Subjective, Objective, Assessment, Plan (SOAP) note format, with a follow-up assessment by the opioid stewardship program’s clinical pharmacy specialist within 72 h to determine whether the recommendation was accepted by the responsible care team.

**Table 1:** High-risk indicators for St. Paul’s hospital’s opioid stewardship program

Patient-related indicators
1. Use of an opioid medication in a patient who is opioid naïve
2. Use of an opioid medication in a patient with personal history of depressive disorder, anxiety disorder and/or post-traumatic stress disorder
3. Use of an opioid medication in a patient greater than 60 years old
Prescription-related indicators
4. High daily dose of an opioid, defined as a prescribed daily dose of 90 morphine milligram equivalent or greater
5. Long duration of opioid prescribing, defined as a patient on opioids on or beyond day 5 of hospitalization
6. Concurrent opioid and sedative prescription
7. Regular dosing of an opioid prescribed for PRN use
8. Long-acting opioid prescriptions within the first 5 days of a patient’s hospital stay
9. Use of parenteral opioids when orders suggest the patient is receiving a normal diet and taking nutrition orally
10. High frequency opioid prescribing, defined as less than 4 h
11. Multiple different opioids prescribed concomitantly for regular and as needed (PRN) use
12. No adjunctive order for non-opioid analgesics including acetaminophen, non-steroidal anti-inflammatory drugs, and/or medications for neuropathic pain (where appropriate)
13. Use of an opioid medication where naloxone administration was required in the last 24 h

### Study sample

For the present study, we restricted to all patient encounters with an active opioid order admitted to St. Paul’s hospital, excluding those followed by emergency department, critical care, palliative care, acute and complex pain services, and addiction medicine. In order to account for pre- and post-opioid stewardship intervention, the study period was from January 2018 to March 2022, with the primary intervention date being January 2020.

### Data sources and measures

We used three hospital data sources: (1) BDM Pharmacy database, which represented all hospital prescribing data prior to the implementation of Cerner (January 2018-November 2019), (2) Sunrise Clinical Manager, which contained scanned copies of all clinical chart notes prior to implementation of Cerner (January 2018-November 2019), and (3) Cerner (November 2019-March 2022) which contains both medication information and clinical records. To ensure data consistency and comparability across the two platforms prior to analysis, two pharmacy team members retrospectively manually coded all variables from the BDM Pharmacy database and Sunrise Clinical Manager to match those available in Cerner.

The primary outcome was the proportion of any high-risk opioid prescribing, based on having at least one of the 10 modifiable prescribing-related factors that were screened for at the time of assessment (Table 1). We also chose three individual prescribing-related factors to assess separately as secondary outcomes in an effort to tease out the potential confounding impact of Cerner on prescribing patterns. These included: 1) > 90MME high daily opioid prescribing; 2) continued opioid use on or after 5 days post-admission; and 3) concurrent opioid-sedative prescribing. We examined > 90MME high daily opioid prescribing given our hypothesis that this indicator would not likely be impacted by Cerner. Opioid use on or after 5 days post-admission and concurrent opioid-sedative prescribing were included as secondary outcomes given our hypothesis that the removal of automatic stop dates in the Cerner system would likely have impacted these two indicators, despite any potential beneficial effects from the opioid stewardship intervention. Ethics was obtained from the Providence Health Care/University of British Columbia Research Ethics Board.

### Statistical analyses

Using interrupted time series (ITS) analysis, we assessed the changes in the level and trend of our primary and secondary outcomes after the implementation of the opioid stewardship program. We utilized segmented regression with autoregressive integrated moving average error models, analyzed over monthly time points. In the present study, we collected serial cross-sectional data on the 15th of every month on every patient encounter who had an active opioid prescription over 49 time points (22 pre-intervention, 25 post-intervention). We used January 2020 as the intervention time point. Data for November and December 2019, and April 2020 were not available due to the electronic system switch to Cerner and an unanticipated pause in the program due to COVID-19, respectively. We assumed that the uptake of Cerner aligned with the opioid stewardship program intervention time point as it was rolled-out only a couple months prior. Given the unit of analysis was patient encounters, we adjusted for the fact that a patient could have multiple encounters in the ITS analysis. We assessed the presence of autocorrelation using the Durbin-Watson test.

We conducted a sensitivity analysis to assess the robustness of our findings, whereby the primary outcome, any high-risk opioid prescribing, was re-defined to remove the two indicators deemed sensitive to the Cerner roll-out (i.e., opioid use on or after day 5 post-admission, concurrent opioid-sedative prescribing). Following this, an ITS analysis was performed.

In additional analyses, we applied a multiple intervention ITS analysis to the concurrent opioid-sedative prescribing outcome, where we accounted for the subsequent interventions that occurred in May 2021 to reverse the unintended consequences of Cerner. As described earlier, Cerner allowed prescribers to order benzodiazepines days before a procedure as part of procedural powerplans (i.e., an electronic order set) with unclear discontinuation plans. In May 2021, efforts to adjust powerplans to ensure benzodiazepines were discontinued post-procedure specifically for gastrointestinal endoscopy, peripherally inserted central catheter (PICC) insertions, and bronchoscopy were implemented. These included review of workflow with the clinical teams responsible for these powerplans, and request to set a 24-hour duration on these procedural powerplans. All p-values were two sided with a significance level of 0.05. All analyses were carried out using Python V.3.12.

## Results

### Study sample

In total, there were 4,622 unique patients who contributed to 5,477 patient encounters with an active opioid order that were included in the present study. Throughout the study duration, on average, 14.0% of eligible patients on any given day were assessed by the opioid stewardship program team via manual chart review (+/- discussion with the patient) and 8.2% received an intervention from the opioid stewardship program team. The average cohort size was 117 patient encounters at each monthly timepoint, with relatively stable demographic characteristics over the study time points. In total, the median age was 65 years (quartile 1–3: 52–75 years), 2,116 (45.9%) were female, and 3,661 (79.2%) were opioid naive (i.e., not prescribed opioids within 30 days prior to their hospital admission). The five most common inpatient hospital services that patients were admitted to included: general internal medicine (16.2%), cardiology (12.1%), cardiac surgery (11.4%), general surgery (10.3%), and geriatric medicine (8.4%).

### Any high-risk opioid prescribing

**Fig. 1:**
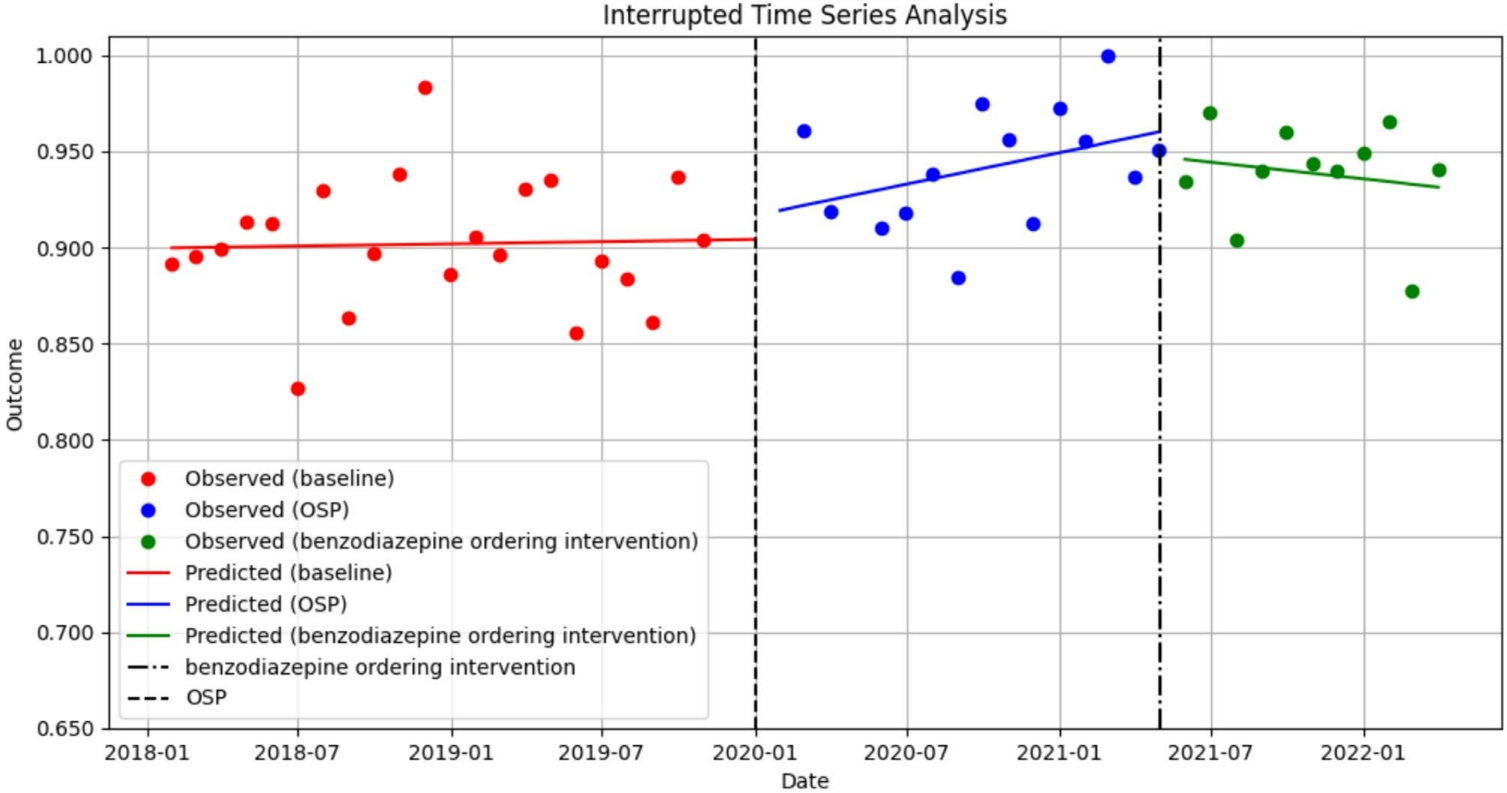
Interrupted time series analysis on the impact of a hospital-based opioid stewardship program (OSP) and subsequent intervention on any high-risk opioid prescribing among active opioid encounters, January 2018– March 2022

The model coefficient estimates for the level and trend changes for the primary and secondary outcomes with their 95% confidence intervals and corresponding *p*-values are available in the Supplementary Table 1. In a multiple intervention ITS, we failed to observe a statistically significant change in either level or trend in any high-risk prescribing post-opioid stewardship program intervention (*p* > 0.05; Fig. 1). Our findings do suggest an initial increase, though not statistically significant (*p* > 0.05), in high-risk opioid prescribing following the opioid stewardship program intervention, which is likely due to removal of automatic stop dates in Cerner and investigated further in our secondary analyses. Multiple intervention ITS accounted for the May 2021 interventions that were subsequently implemented to specifically address the co-prescribing of opioids and sedatives, though again, failed to find any significant change in level or trend of overall high-risk opioid prescribing (*p* > 0.05).

In sensitivity analyses (shown in Supplementary Tables 2 and Supplementary Fig. 2), we ran an ITS analysis using an alternate outcome of any high-risk opioid prescribing, which removed two indicators that were sensitive to the Cerner roll-out (i.e., opioid use on or after day 5 post-admission, concurrent opioid-sedative prescribing). While we did not observe a statistically significant change in level and trend of high-risk opioid prescribing using this definition, we showed a decreasing slope change post-opioid stewardship intervention that was trending towards statistical significance (estimate: -0.006, 95%CI: -0.012, 0.000, *p* = 0.060).

### High daily dose opioid prescribing

**Fig. 2:**
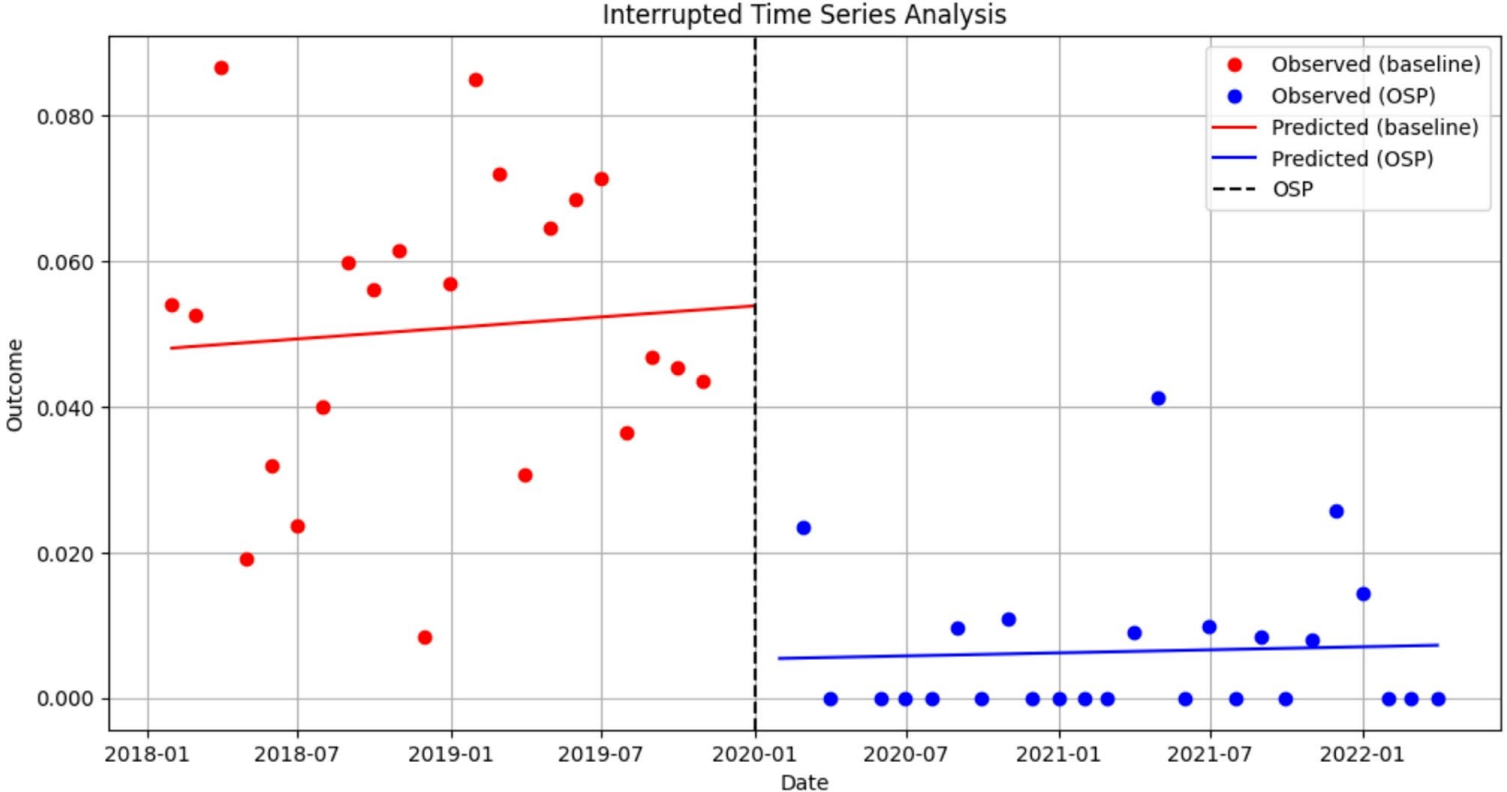
Interrupted time series analysis on the impact of a hospital-based opioid stewardship program (OSP) on high daily dose of opioid prescribing among active opioid encounters, January 2018– March 2022

Following the examination of our primary outcome, any high-risk opioid prescribing, we were interested in separately assessing indicators that may not have been impacted by the simultaneous Cerner roll-out. In Fig. 2, the ITS results showed an immediate reduction in the level of high daily dose opioid prescribing post-opioid stewardship program intervention. The estimated level dropped by 0.044 (95%CI: -0.082, -0.006, *p* = 0.025).

### Long duration of opioid use

**Fig. 3:**
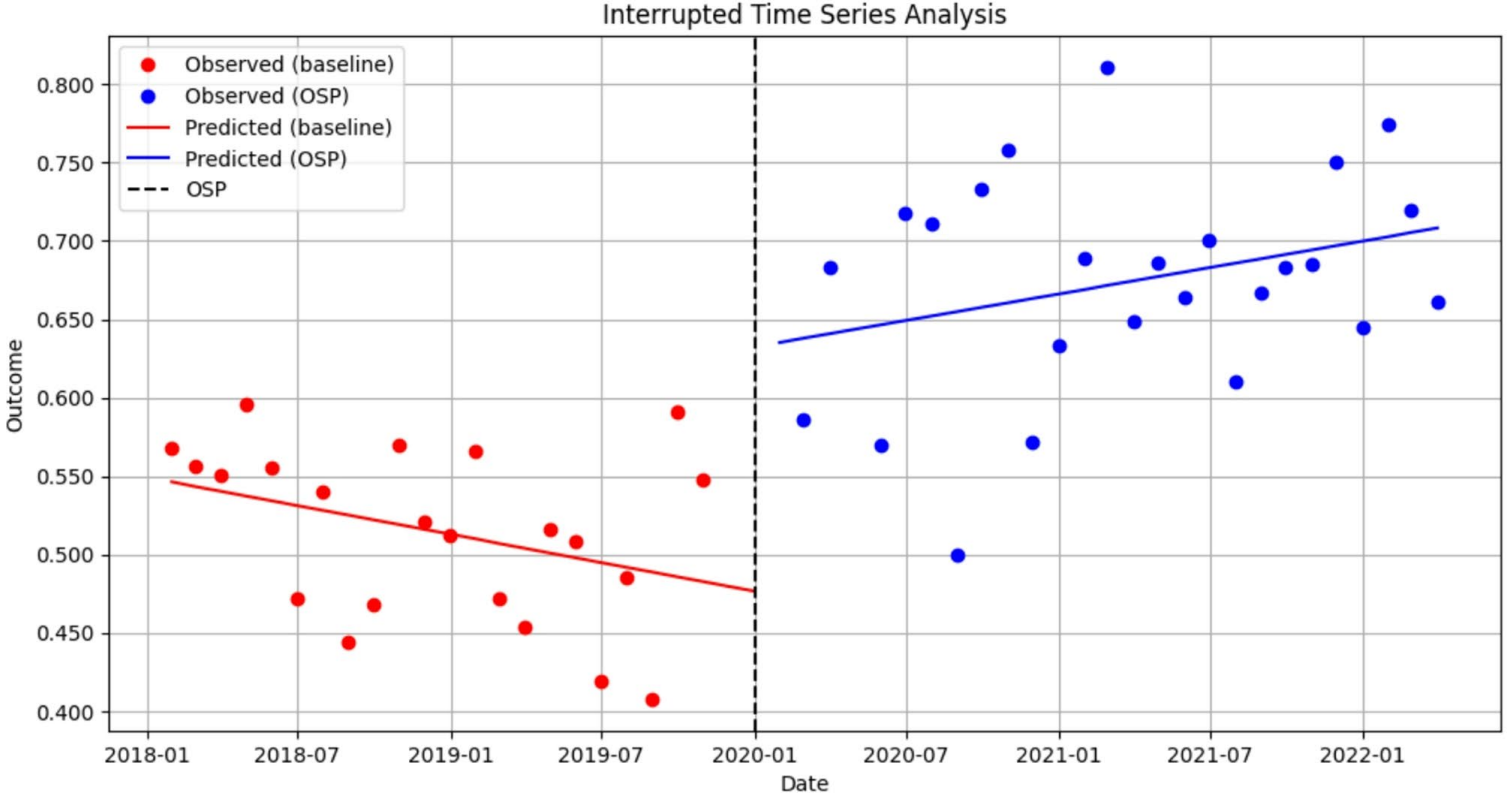
Interrupted time series analysis on the impact of a hospital-based opioid stewardship program (OSP) on long duration of opioid prescribing among active opioid encounters, January 2018– March 2022

We hypothesized that continued opioid use on or after 5 days post-admission would have been impacted by the Cerner system, given the removal of automatic stop dates for opioid orders, despite any beneficial impacts that would have been observed by the opioid stewardship program. As indicated in Fig. 3, no statistically significant changes were seen in the proportion of patients who continued to be prescribed opioids on or after day 5 of their hospital admission following implementation of the opioid stewardship program (*p* > 0.05). However, we observed an increase in the trend post-implementation (estimate: 0.06; 95%CI: 0.000, 0.011, *p* = 0.034).

### Concurrent opioid-sedative prescribing

**Fig. 4:**
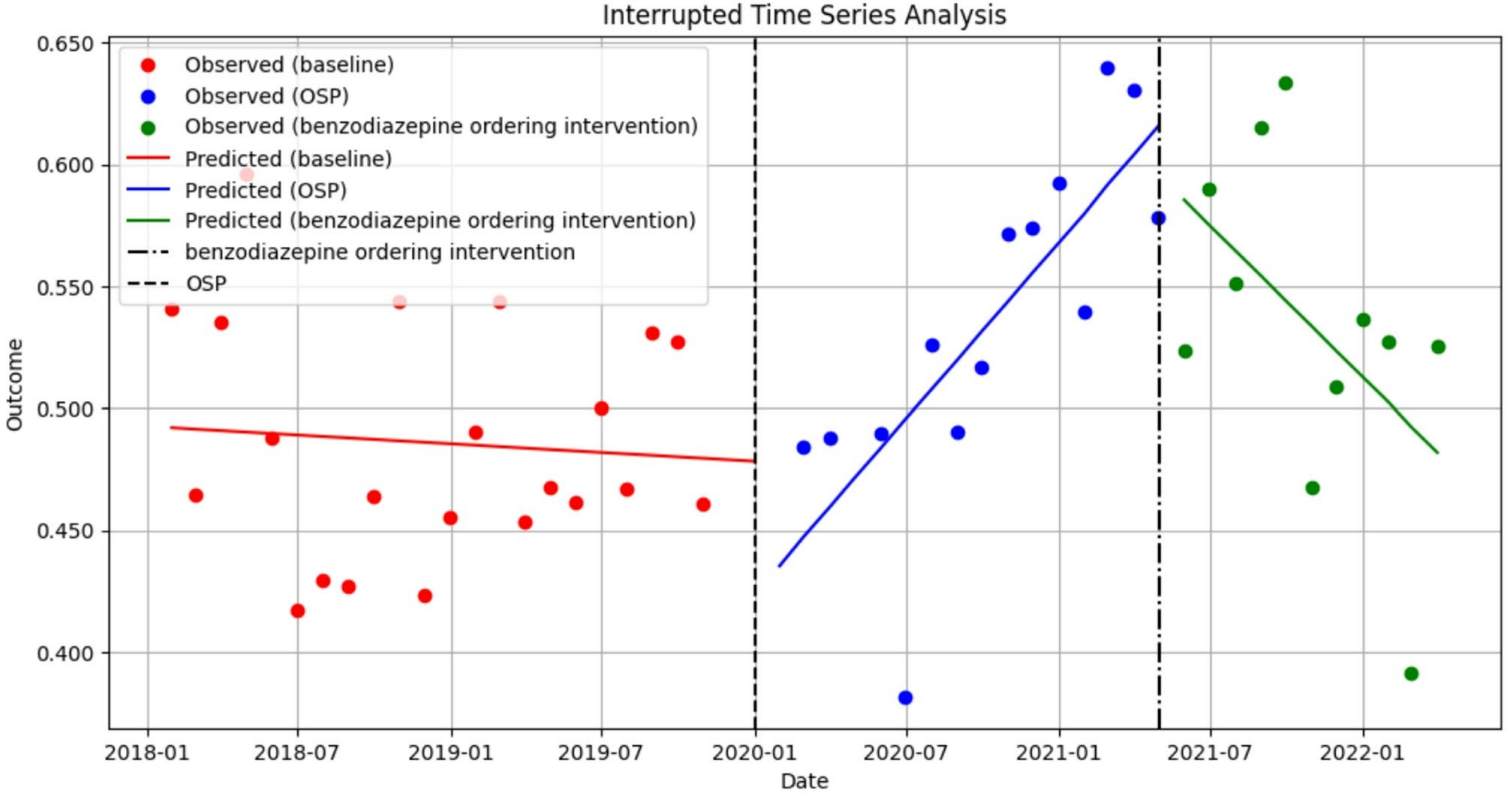
Interrupted time series analysis on the impact of a hospital-based opioid stewardship program (OSP) on concurrent opioid-sedating prescribing among active opioid encounters, January 2018– March 2022

Pre-intervention, 49.3% of hospitalized patients on average were concurrently prescribed an opioid and sedative medication. Shown in Fig. 4, our findings suggest that the opioid stewardship program initially reduced concurrent opioid-sedative prescriptions (estimate: -0.357; 95%CI: -0.579, -0.136, *p* = 0.002), with a subsequent increase over time (estimate: 0.013; 95%CI: 0.005, 0.020, *p* = 0.001). Interventions implemented in May 2021 reversed this trend, reducing both the level (estimate: 0.874; 95%CI: 0.374, 1.375, *p* = 0.001) and slope (estimate: -0.022, 95%CI: -0.034, -0.011, *p* < 0.001) of concurrent prescriptions.

## Discussion

In the present study, we showed that the simultaneous implementation of a hospital-based opioid stewardship program and EHR with CPOE system resulted in mixed outcomes. Our findings indicate that the opioid stewardship program effectively decreased high-dose opioid prescriptions. However, the benefits of opioid stewardship were not observed for other outcomes that may have been influenced by an EHR with CPOE (i.e., long duration of opioid use, concurrent opioid-sedative prescribing). Regardless, the opioid stewardship program enabled the team to observe and monitor opioid prescribing trends over time, and demonstrated its agility by promptly addressing unintended consequences through interventions aimed at reducing unnecessary concurrent prescribing of opioids and sedatives.

Our findings add to the existing literature showing the potential benefits of a hospital-based opioid stewardship program by demonstrating a reduction in the proportion of patients who were being prescribed high daily doses of opioids. While we know of no studies that have examined the impact of an opioid stewardship program specifically on high-dose prescribing, indeed, our findings are consistent with previous studies that have shown reductions in the amount of opioid prescriptions in postsurgical and emergency settings [24, 25]. A growing body of research has demonstrated the negative consequences of high dose opioid prescribing in both hospital and community settings. For example, a population-level study conducted in Ontario, Canada found that individuals who were prescribed opioid daily doses exceeding 200 MME had a 2.97 times the hazard of overdose compared to those prescribed 20 MME or less daily [32]. Based on our findings, strategies like opioid stewardship that incorporate audit-and-feedback mechanisms alongside consultations should be considered in hospitals with high rates of high-dose opioid prescribing.

Unexpectedly, the simultaneous roll-out of an EHR with CPOE may have complicated our findings, demonstrating how much impact systems-level changes can have on prescribing patterns. Specifically, our study showed that the proportion of patients who were exposed to a long duration of opioid prescribing increased both in level and trend after the implementation of the opioid stewardship program, which was likely attributed to the removal of automatic stop dates for opioids in the EHR system. We also found that the proportion of patients who were co-prescribed opioids and sedative medications increased post-intervention. Prior to the EHR with CPOE implementation, benzodiazepine orders were only active for the day of a particular procedure, whereas post-system implementation, there was the option of ordering these medications days before a procedure, without clear guidance on whether the clinic or the ward would discontinue the final order set. Our findings are in line with previous studies that have discussed how computerized order entry systems for medications in hospitals do not necessarily reduce errors, but rather may introduce different types of errors into the healthcare system [33–35]. Of relevance, medication discontinuation failures were common among studies that examined the unintended consequences of computerized orders [33, 36]. Our findings further highlight the fact that decisions around automatic stop dates seem to have a negative impact on opioid prescribing risk indicators. Taken together, these results lend evidence to the need to monitor the potential risks that introducing EHR systems may have on prescribing patterns in acute care settings. Moreover, hospitals that are implementing new EHRs with CPOE need to carefully consider how they apply automatic stop dates within their system.

Importantly, we found that the opioid stewardship program proved able to monitor opioid prescribing over time and swiftly respond to these unintended system-level consequences introduced by the EHR with CPOE. In particular, monitoring of opioid prescribing allowed the clinical team to adjust powerplans appropriately to ensure appropriate benzodiazepine discontinuation. As shown, these interventions, which were introduced in May 2021, coincided with a subsequent decline in the proportion of patients being co-prescribed opioids and sedative medications. Opioid stewardship has the potential to make individualized clinical recommendations as well as timely address system-level changes in an effort to create sustained improvement when issues are identified. Our findings point to the value of opioid stewardship in responding to unintended consequences in a time-sensitive manner, and the potential need for other stewardship programs focused on high-risk medications (e.g., anticoagulants) operating alongside any health system changes in how medications are prescribed [37, 38]. 

There are some limitations to this study. First, St. Paul’s Hospital provides care to a unique urban population with a high prevalence of substance use and psychiatric conditions; as such, our findings may not be generalizable to other hospitals. Second, while our goal was to assess the impact of an opioid stewardship program on high-risk opioid prescribing, we encountered difficulty in isolating the effects of the simultaneous implementation of the EHR with CPOE. Third, we were not able to find an appropriate control population who did not receive the intervention to conduct a comparative ITS. While we were unable to directly link individual prescribing behaviours to the opioid stewardship program, our goal was to impact overall trends in hospital prescribing of opioids, which we believe we were able to demonstrate. Lastly, due to the large number of patients identified as potentially receiving high risk opioids in the hospital, the clinical team needed to prioritize their efforts. As a result, they were unable to reach and provide recommendations to all eligible patients on any given day. Additionally, patients may have been eligible to receive an intervention on multiple days but may have been reviewed the day before or after the extracted cross-sectional monthly time point, hence the estimates of eligible patients reviewed and interventions recommended may have slightly underestimated the overall proportion of patient engagement with the program. This capacity limitation may have diminished the overall observed effectiveness of the opioid stewardship program.

In summary, we found mixed results on the impact of a hospital-based audit-and-feedback opioid stewardship program on reducing high-risk opioid prescribing. Our varied findings can likely be attributed to the concurrent implementation of an electronic health record system with computerized medication order entries, which may have impacted some high-risk prescribing indicators. Nevertheless, our research underscores the importance of opioid stewardship initiatives in monitoring prescription practices within acute care environments by highlighting their capacity to identify and respond to unintended consequences resulting from broader systemic changes.

## Electronic supplementary material

Below is the link to the electronic supplementary material.

Supplementary Material 1

## Data Availability

No datasets were generated or analysed during the current study.
