# Supplementary material for: Evaluation of a hospital-based opioid stewardship program on high-risk opioid prescribing in a Canadian setting: an interrupted time series analysis
Source: Addict Sci Clin Pract. 2025 Jun 6;20:47. doi: 10.1186/s13722-025-00574-x (PMC12143091; doi:10.1186/s13722-025-00574-x)
Supplement: Supplementary file 1 — Supplementary Material 1 [file 13722_2025_574_MOESM1_ESM.docx]

| **Supplementary Table 1. Model coefficients of level and trend changes on high-risk opioid prescribing in interrupted time series analysis** | | | |
| --- | --- | --- | --- |
|  | **Estimate** | **95% confidence interval** | **p-value** |
| *Any high-risk opioid prescribing* | | | |
| **Level (baseline)** | 0.900 | 0.871, 0.928 | <0.001 |
| **Trend (baseline)** | 0.000 | -0.002, 0.002 | 0.859 |
| **Level change (OSP)** | -0.049 | -0.187, 0.090 | 0.484 |
| **Trend change (OSP)** | 0.003 | -0.002, 0.007 | 0.270 |
| **Level change (intervention)** | 0.154 | -0.160, 0.468 | 0.327 |
| **Trend change (intervention)** | -0.004 | -0.012, 0.003 | 0.258 |
| *High daily dose of opioid prescribing* | | | |
| **Level (baseline)** | 0.048 | 0.033, 0.062 | <0.001 |
| **Trend (baseline)** | 0.000 | -0.001, 0.001 | 0.647 |
| **Level change (OSP)** | -0.044 | -0.082, -0.006 | 0.025 |
| **Trend change (OSP)** | -0.000 | -0.002, 0.001 | 0.797 |
| *Long duration of opioid prescribing* | | | |
| **Level (baseline)** | 0.550 | 0.495, 0.604 | <0.001 |
| **Trend (baseline)** | -0.003 | -0.007, 0.001 | 0.150 |
| **Level change (OSP)** | 0.015 | -0.130, 0.160 | 0.832 |
| **Trend change (OSP)** | 0.006 | 0.000, 0.011 | 0.034 |
| *Concurrent opioid-sedative prescribing* | | | |
| **Level (baseline)** | 0.493 | 0.447, 0.538 | <0.001 |
| **Trend (baseline)** | -0.001 | -0.004, 0.003 | 0.729 |
| **Level change (OSP)** | -0.357 | -0.579, -0.136 | 0.002 |
| **Trend change (OSP)** | 0.013 | 0.005, 0.020 | 0.001 |
| **Level change (intervention)** | 0.874 | 0.374, 1.375 | 0.001 |
| **Trend change (intervention)** | -0.022 | -0.034, -0.011 | <0.001 |

| **Supplementary Table 2. Interrupted time series analysis on the impact of a hospital-based opioid stewardship program and subsequent intervention on any high-risk opioid prescribing among active opioid encounters, sensitivity analysis with alternate definition** | | | |
| --- | --- | --- | --- |
|  | **Estimate** | **95% confidence interval** | **p-value** |
| **Level (baseline)** | 0.667 | 0.603, 0.731 | <0.001 |
| **Trend (baseline)** | 0.004 | -0.001, 0.008 | 0.145 |
| **Level change (OSP)** | 0.118 | -0.050, 0.287 | 0.164 |
| **Trend change (OSP)** | -0.006 | -0.012, 0.000 | 0.060 |

| **Supplementary Figure 1. Interrupted time series analysis on the impact of a hospital-based opioid stewardship program (OSP) on any high-risk opioid prescribing among active opioid encounters, sensitivity analysis with alternate definition**  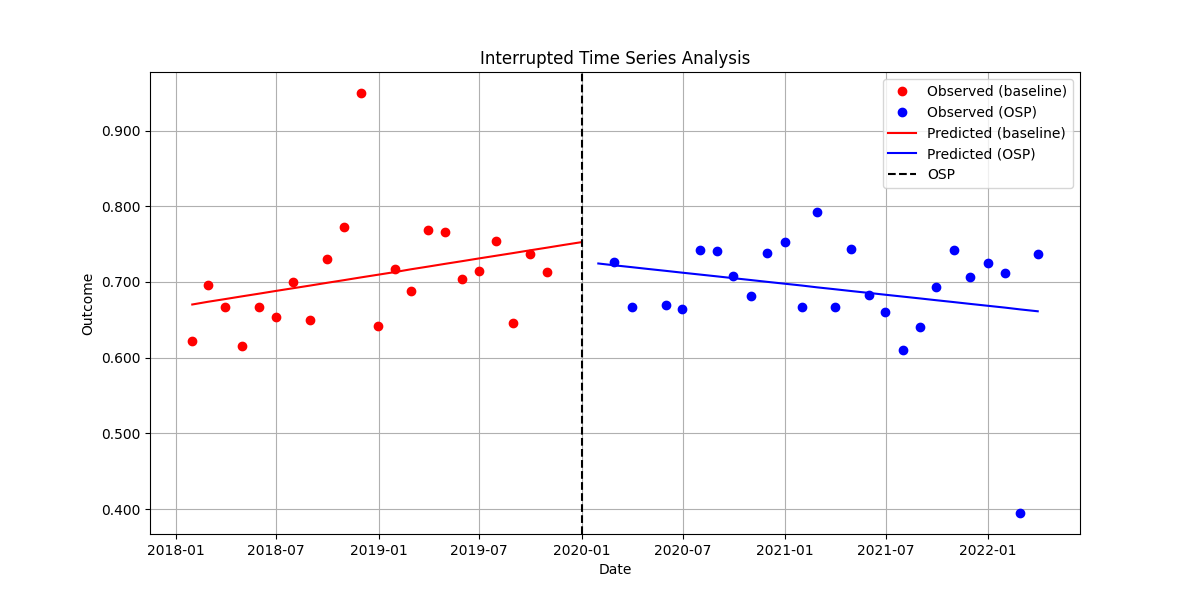 |
| --- |
